# Effect of Chlorhexidine Digluconate in Early Wound Healing of Human Gingival Tissues. A Histological, Immunohistochemical and Biomolecular Analysis

**DOI:** 10.3390/antibiotics10101192

**Published:** 2021-10-01

**Authors:** Andrea Pilloni, Simona Ceccarelli, Daniela Bosco, Giulia Gerini, Cinzia Marchese, Lorenzo Marini, Mariana A. Rojas

**Affiliations:** 1 Department of Oral and Maxillofacial Sciences, Section of Periodontics, Sapienza University of Rome, Via Caserta 6, 00161 Rome, Italy; andrea.pilloni@uniroma1.it (A.P.); lorenzo.marini@uniroma1.it (L.M.); 2 Department of Experimental Medicine, Sapienza University of Rome, Viale Regina Elena 324, 00161 Rome, Italy; simona.ceccarelli@uniroma1.it (S.C.); giulia.gerini@uniroma1.it (G.G.); cinzia.marchese@uniroma1.it (C.M.); 3 Department of Radiological, Oncological and Pathological Sciences, Sapienza University of Rome, Viale del Policlinico 155, 00161 Rome, Italy; daniela.bosco@uniroma1.it

**Keywords:** apoptosis, chlorhexidine digluconate, gingiva, human biopsy, wound healing

## Abstract

Chlorhexidine digluconate (CHX) is considered the gold standard for oral cavity antiseptic treatment. Nevertheless, several in vitro studies have reported detrimental effects in oral tissue repair. The aim of the present study was to evaluate the in vivo effect of post-surgical CHX mouth rinse on gingival tissue (G) 24 h after injury. G biopsies were obtained in three patients 24 h after surgery with the indication of post-surgical 0.12% CHX use and were compared with those obtained from the same patients without any antiseptic use. Changes in collagen production, cell proliferation, and apoptosis were examined by histological and Ki-67/P53 immunohistochemical analysis. Fibrotic markers (COL1A1, αSMA), proapoptotic protein (BAX) expression, and wound healing-related gene modulation (RAC1, SERPINE1, TIMP1) were analyzed by quantitative real-time PCR analysis. CHX was able to reduce cellular proliferation and increase collagen deposition, proapoptotic molecule and fibrotic marker expression, and myofibroblast differentiation, reduce expression of RAC1 and trigger expression of SERPINE1 and TIMP1, showing “scar wound healing response” pattern. This study assessed for the first time the in vivo effects of CHX on gingival tissue. The demonstration of a CHX-induced fibrotic transformation, leading to scar repair, supports the need for new post-surgical clinical protocols based on a strategic and personalized use of CHX.

## 1. Introduction

Wound healing is a particularly complex physiological process that depends on multiple factors [1]. The presence of oral biofilm, the main etiological factor of periodontal and peri-implant diseases, may jeopardize the repair process [2]. For this reason, especially after surgical procedures in which mechanical plaque control cannot be performed, the reduction of plaque accumulation by means of antimicrobial agents is extremely important [3].

Chlorhexidine digluconate (CHX), a bisbiguanide broad-spectrum antiseptic with antibacterial action, is widely used as a therapeutic agent in periodontology. Numerous studies have demonstrated the ability of CHX to reduce oral biofilm deposition [4,5,6]. Moreover, by penetrating biofilms, CHX shows a bactericidal action [7], reaching a substantivity of 12 h [8].

Although different effects have been reported based on a variety of available concentrations, a study conducted by Jones in 1997 concluded that twice daily rinses with 15 mL of 0.12% CHX are enough for effective plaque control in the oral cavity [9].

However, side effects of CHX mouth rinses, such as desquamation of the oral mucosa, soreness, increased calculus formation, and tooth discoloration have already been reported in the literature, suggesting strict control in their use and recommending only for short periods [10].

In a recent systematic review [11], the authors concluded that CHX helps in reducing biofilm formation and gingival inflammation after periodontal and implant surgery and that less concentrated formulations (e.g., 0.12%) should be indicated in order to reduce the adverse effects.

Due to the above mentioned bactericidal and bacteriostatic activities [4,5,6] and to the absence of toxic systemic effects reported [12], CHX has been considered the gold standard for antiseptic treatment of the oral cavity [9]. Nevertheless, a recent in vitro study evaluating the impact of CHX use in controlling oral biofilms showed an initial drop in biofilm bacterial cell concentration followed by a quick recovery after its use. Therefore, the authors concluded that CHX can be ineffective in maintaining oral health since it presents a temporal effect and, as a broad-spectrum antiseptic, it can also affect the endogenous oral microbiota, increasing the risk of microbial dysbiosis, leading in turn to the development of oral diseases [13].

Furthermore, since the 1970s, several studies have reported noxious effects on many different cells as macrophages [14], leucocytes [15], and skin epithelial cells [16]. Bassetti and Kallenberger in 1980 [17] through an animal experimental model demonstrated that intensive post-surgical rinsing with high concentrations of CHX could delay and impair the wound repair process. In addition, many recent studies showed cytotoxic effects in human periodontal tissues cells, such as gingival epithelial cells [18], gingival fibroblasts [19,20,21], bone [22], and periodontal ligament cells [23].

Faria et al. [20] observed that CHX induces apoptosis of cultured fibroblasts at lower concentrations and necrosis at higher concentrations. Mariotti and Rumpf [19] postulated that CHX can reduce both collagen and non-collagen protein production and proliferation of human gingival fibroblasts (HGFs), even at very low concentrations, and this negatively affects the wound healing process. This was confirmed in a recent in vitro study in which cells were exposed to a concentration diluted 100-fold when compared to the current use in clinical practice [24].

Another recent in vitro study using HGFs showed that a CHX concentration ≥0.04% inhibits cell proliferation, affects cells morphology, and induces apoptosis. These effects are concentration and time-dependent. The authors concluded that post-surgical applications of CHX should be limited [25].

All the above-mentioned in vitro studies indicate that CHX is not harmless to oral tissues, mainly in the wound healing process. However, it is important to highlight that in vitro assays cannot fully represent the oral environment as a whole and this could be a limitation [26].

Chen et al. [27] demonstrated that the main transcriptional changes in the wound healing process occur in the first 12–24 h. In fact, we observed significant changes in myofibroblast differentiation, fibrotic markers, and wound healing gene expression of oral soft tissue derived-fibroblasts 24 h after surgery, when compared to baseline [28,29]. In addition, it was demonstrated that until the first 24 h the biofilm is primarily populated by gram-positive cocci, and gram-negative anaerobic bacteria rapidly increase and predominate after 48 h [30,31].

Considering all the aforementioned, the immediate, post-surgical use of CHX might not be necessary. This could be of beneficial effect on the healing process, since the most important changes in tissue repair occur in the very early stages.

To date, no in vivo study has been conducted evaluating CHX effects on gingival tissue behavior in the early wound healing process.

Therefore, the aim of the present study was to evaluate the in vivo effect of post-surgical CHX mouth rinse on the gingival tissues, 24 h after injury. Our hypothesis was that CHX impairs the wound healing potential by: (1) reducing the proliferation ability, (2) increasing cell apoptosis, fibrotic marker expression and myofibroblast differentiation and (3) modifying early wound healing-related gene expression, as determined by histological, immunohistochemical and biomolecular analyses of human gingival biopsies.

## 2. Results

### 2.1. CHX Post-Surgical Mouth Rinse Increases Fibrotic Marker Expression and Myofibroblast Differentiation

Myofibroblast activation and collagen deposition are key events in physiological and pathological tissue repair.

**Figure 1:**
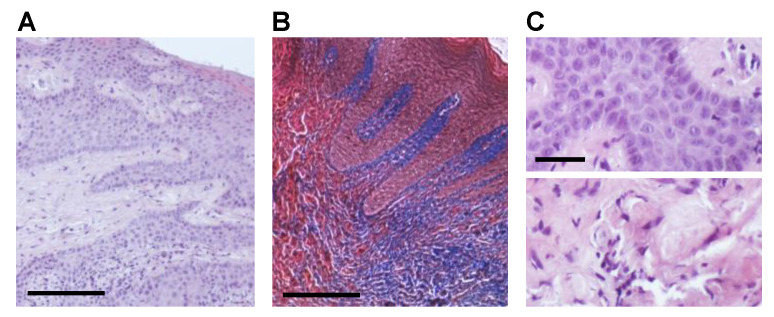
Histological characterization of gingival biopsies. (**A**) Representative photomicrograph of sections of gingival biopsies showing elongated and branched epithelial ridges and subjacent chorion full of a dense and homogeneous structure of collagen bundles. HE staining, scale bar 100 μm. (**B**) Representative photomicrograph of sections of gingival biopsies showing collagen bundles in the deep chorion (blue). Trichromic Masson staining, scale bar 100 μm. (**C**) Representative photomicrographs of histological alterations observed in CHX biopsies, such as enlarged and polymorphic nuclei in the epithelial layer (upper panel) and enhanced fibrosis in the deep chorion (lower panel). HE staining, scale bar 25 μm.

To identify the effect of CHX treatment on the phenotype of fibroblasts involved in collagen synthesis, we analyzed gingival biopsies of three patients subjected or not to CHX mouth rinses in the 24 h between surgical intervention and biopsy collection. HE staining revealed in both the NT and CHX group a thick gingival mucosa, with deep and branching epithelial ridges, partly joined by epithelial bridges. Subjacent chorion was full of collagen bundles, appearing as a dense and homogeneous structure (Figure 1A). Collagen deposition was further revealed with Masson’s trichrome staining (Figure 1B). As for CHX group, HE staining showed the presence of enlarged, polymorphic and polymetric nuclei, indicative of activated cells, in the epithelial layer (Figure 1C, upper panel), and a more extensive fibrosis in the chorion (Figure 1C, lower panel).

**Figure 2:**
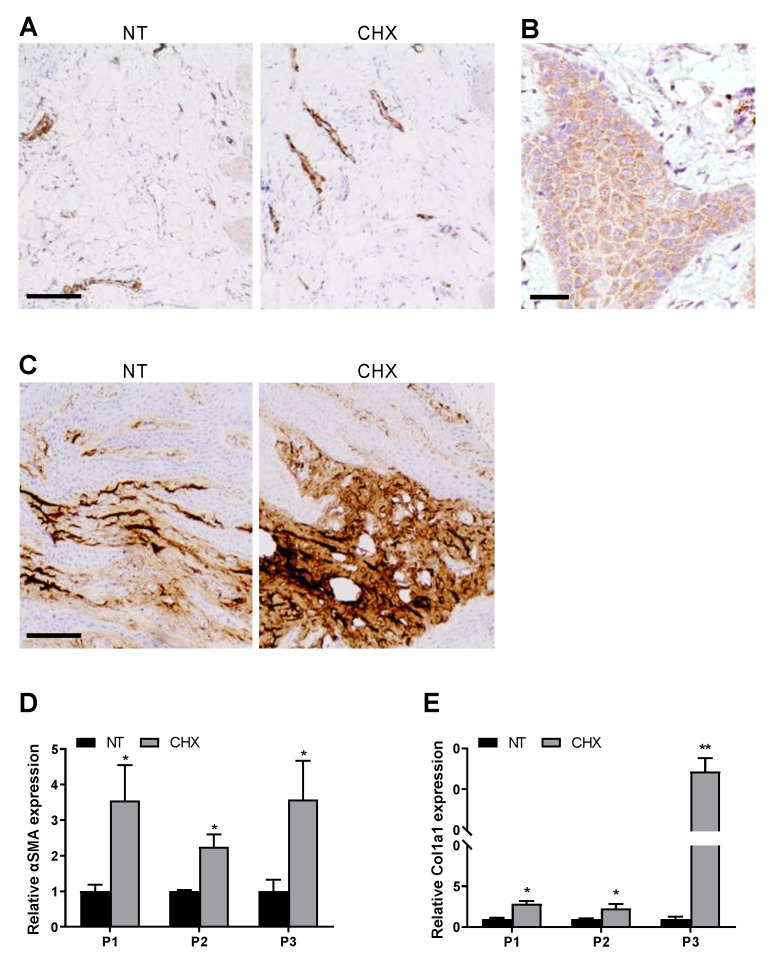
CHX increases protein and mRNA expression of fibrotic markers αSMA and Col1A1 in gingival tissues. (**A**) Representative photomicrographs of sections of NT and CHX gingival biopsies stained with anti-αSMA. Scale bar 100 μm. (**B**) Representative photomicrograph of cytoplasmic staining for αSMA in the epithelial layer observed in CHX biopsies. Scale bar 25 μm. (**C**) Representative photomicrographs of sections of NT and CHX gingival biopsies stained with anti-Col1a1 antibodies. Scale bar 100 μm. (**D**,**E**) Quantitative real-time PCR analysis of αSMA (**D**) and Col1a1 (**E**) mRNA expression in NT and CHX biopsies of three patients. Relative mRNA levels are shown as fold value of the NT levels. mRNA levels were normalized to GAPDH mRNA expression. Each experiment was performed in triplicate. Error bars represent standard deviations. * *p* < 0.05 and ** *p* < 0.005 vs. NT.

Afterwards, the expression levels of fibrosis markers were analyzed with IHC staining. We incubated serial sections of each biopsy belonging to the two groups (NT and CHX) with the following antibodies: anti-αSMA, anti-Col1a1, and anti-vimentin. For αSMA, normal vessel smooth muscle immunoreactivity was used as an internal positive control, while αSMA-positive stromal cells, showing cytoplasmic immunostaining, were considered to be myofibroblasts. NT samples showed an extremely weak positivity in the mesenchymal cells, while cells of blood vessels were labeled. In the CHX group, we noted a higher number of blood vessels in the chorionic papillae and the deep chorion compared to NT samples (Figure 2A), and we also observed the presence of cells with cytoplasmic positivity localized in the basal epithelial layer, particularly in the deep and prickle cell layers (Figure 2B).

As for the fibrotic marker Collagen 1a1 (Col1a1), its expression was localized in the subepithelial layer, and it was significantly higher in CHX biopsies with respect to the NT group (Figure 2C).

Immunostaining for vimentin, specific for cells of mesenchymal origin, showed a few positive cells concentrated mainly in the subepithelial layer. We observed no significant differences both in the amount of positive cells and in their location between samples from the NT and CXH groups.

The semiquantitative evaluation for αSMA, Col1a1, and vimentin staining intensity is reported in Table 1.

The expression of αSMA and Col1a1 was also assessed at mRNA level by qRT-PCR analysis in gingival biopsies of three patients subjected or not to CHX mouth rinses in the 24 h between surgical intervention and biopsy collection. Our results confirmed a significant increase in αSMA expression in the CHX biopsies of all the three patients (3.6, 2.3, and 3.6-fold, respectively) (Figure 2D). The same trend was observed for Col1a1, with a consistent increase in the CHX biopsies of all patients (2.9, 2.3, and 34.4-fold, respectively) (Figure 2E).

### 2.2. CHX Influences the Expression of Key Genes Involved in Early Wound Healing

**Figure 3:**
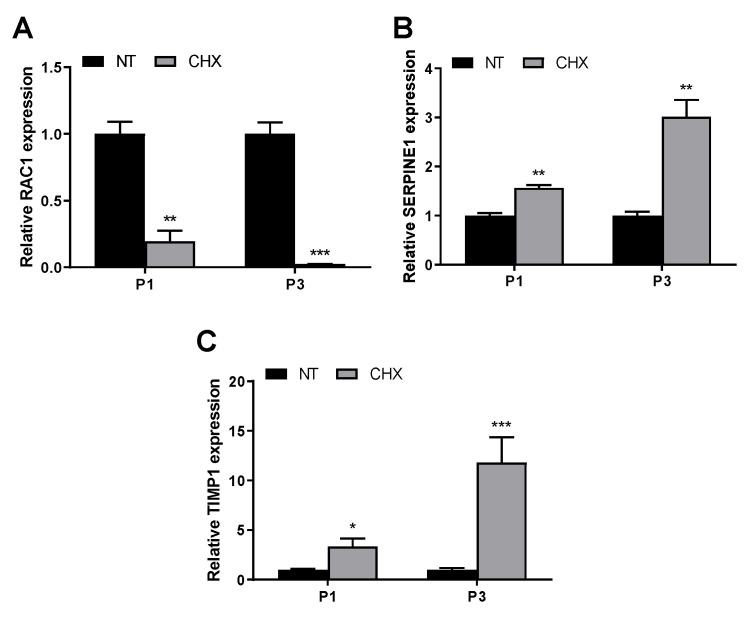
CHX modulates mRNA expression of genes involved in early wound healing. Quantitative real-time PCR analysis of RAC1 (**A**), SERPINE1 (**B**) and TIMP1 (**C**) mRNA expression in NT and CHX biopsies of two patients. Relative mRNA levels are shown as fold value of the NT levels. mRNA levels were normalized to GAPDH mRNA expression. Each experiment was performed in triplicate. Error bars represent standard deviations. * *p* < 0.05, ** *p* < 0.005, and *** *p* < 0.0005 vs. NT.

We then investigated the effect of CHX on the expression of some previously shown genes in playing a role in the early wound healing process [29], in two out of the three enrolled patients (since in one of the patients the material obtained with the biopsy was not enough to carry out the analysis). We first evaluated RAC1, a member of the Rho family of small GTPases that promotes healing and that has been previously shown to increase in gingival tissue, 24 h after injury [29]. Interestingly, we observed a significant downmodulation of RAC1 expression at 24 h in CHX biopsies of both patients (0.2 and 0.02-fold, respectively) (Figure 3A), thus suggesting that CHX might impair gingival wound healing. The other two genes that play a role in regulating scar formation in oral tissues, SERPINE1 and TIMP1, were evaluated. Such genes, involved in collagen deposition and fibrosis, were previously shown to remain stable in gingival tissue at 24 h after injury [29]. In our study, we observed an increase of SERPINE1 and TIMP1 in CHX biopsies of both patients (1.6 and 3.0-fold for SERPINE1; 3.4 and 11.8-fold for TIMP1, respectively; Figure 3B,C).

### 2.3. CHX Increases the Expression of Apoptotic Markers and Reduces the Proliferative Ability of Gingival Cells

**Figure 4:**
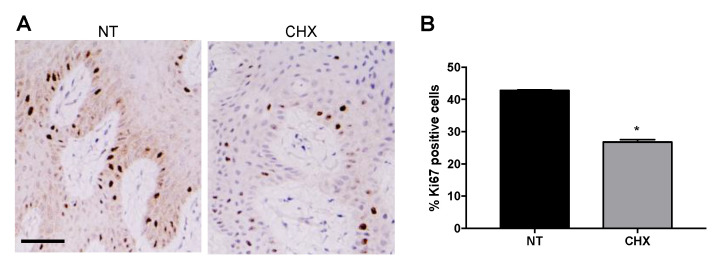
CHX shows an antiproliferative effect in gingival tissues. (**A**) Representative photomicrographs of sections of NT and CHX gingival biopsies stained with anti-Ki67 antibodies. Scale bar 50 μm. (**B**) Mean percentage of Ki67 immunopositive cells. * *p* < 0.05 vs. NT.

In order to understand the molecular events underlying the effect of CHX on early gingival wound healing, the expressions of proteins related to proliferation and apoptosis were examined by IHC analysis. As compared with the NT group, the Ki67 proliferation marker was significantly downregulated in the CHX group (Figure 4A), as indicated by the percentage of stained nuclei reported in Figure 4B (26.8% vs. 42.8% of NT, * *p* < 0.05).

**Figure 5:**
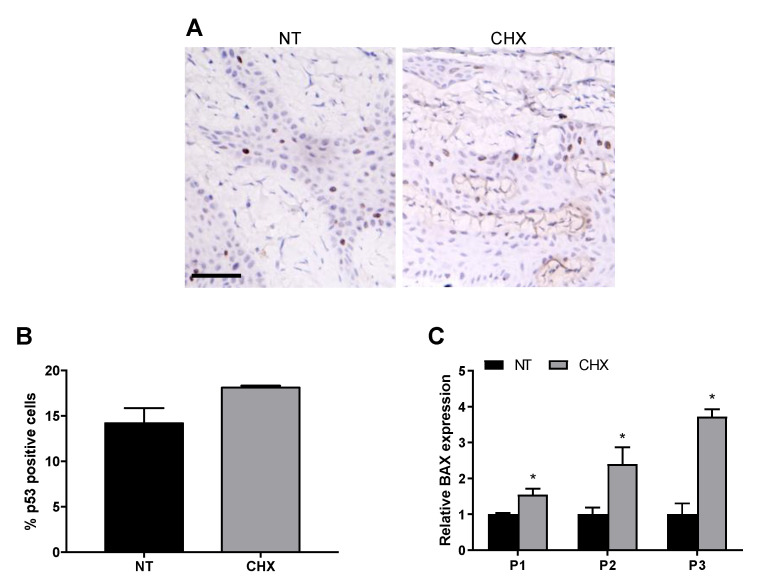
CHX shows a proapoptotic effect in gingival tissues. (**A**) Representative photomicrographs of sections of NT and CHX gingival biopsies stained with anti-p53 antibodies. Scale bar 50 μm. (**B**) Mean percentage of p53 immunopositive cells. (**C**) Quantitative real-time PCR analysis of BAX mRNA expression in NT and CHX biopsies of three patients. Relative mRNA levels are shown as fold value of the NT levels. mRNA levels were normalized to GAPDH mRNA expression. Each experiment was performed in triplicate. Error bars represent standard deviations. * *p* < 0.05 vs. NT.

Therefore, we assessed if the reduced proliferation could be accompanied by an induction of apoptosis. To this aim, we evaluated the expression of the tumor suppressor gene p53, a key regulator of cell death under multiple physiological and pathological conditions. In our in vivo model, IHC analysis showed that p53 expression was slightly higher in the CHX group (Figure 5A), with a modest but not statistically significant increase of the percentage of stained nuclei in CHX samples (18.1% vs. 14.2% of NT, Figure 5B).

Interestingly, when analyzing the expression of the proapoptotic BAX protein in gingival tissue by real time PCR, we found a significantly higher expression of BAX in the CHX biopsies of all the enrolled patients (1.5, 2.4 and 3.7-fold, respectively) (Figure 5C), thus indicating a potential p53-independent proapoptotic effect of CHX post-surgical treatment on gingival tissue.

## 3. Discussion

Chlorhexidine is considered as the gold standard in the antiseptic treatment of the oral cavity [9]. Nevertheless, a time and dose-dependent cytotoxic effect of CHX in human fibroblasts has been demonstrated in previous in vitro studies [19,26,34], delaying wound healing or increasing wound dehiscence rates [35,36].

The present study was designed to investigate the in vivo effect of post-surgical 0.12% CHX mouth rinse in the early phase of gingival tissue repair to understand its role on cell behavior, including (1) proliferation, (2) apoptosis, (3) fibrotic marker expression, (4) myofibroblast differentiation, and (5) early wound healing-related gene expression through a histological, immunohistochemical and biomolecular analysis of human gingival biopsies. All these processes are involved in the soft tissue wound healing response after surgical procedure.

Our findings demonstrate that, 24 h after injury, CHX is able to (a) reduce cell proliferation and increase the expression of proapoptotic molecules, (b) increase fibrotic marker expression and myofibroblast differentiation, (c) reduce expression of RAC1 gene, characterizing keratinocyte migration and proliferation, and the ability of the oral wound to respond to stress, and (d) trigger expression of SERPINE1 and TIMP1, which regulate scar wound healing.

In our in vivo experimental setting, we observed that Ki67 proliferation marker was significantly downregulated in the CHX group compared with the NT group, confirming the anti-proliferative effects of CHX in gingival tissue in vivo, in agreement with those obtained in vitro by other authors [19,25,26,37]. Many cytotoxic agents modulate the balance between cell proliferation and cell death [38]. Cell death can occur through different pathways that can culminate in autophagy, necrosis, or apoptosis [25]. These mechanisms may play an important role in the scarring response. In fact, the ability of apoptotic cells to induce myofibroblast differentiation and proliferation has been reported [39,40].

Gianelli et al. [41] reported that after 1 min treatment, nearly 50% of fibroblastic and endothelial cells treated with 0.12% CHX exhibited apoptotic nuclei. Regarding this concern, some clarifications need to be pointed out as follows. In the present work, our goal was not to study the amount of apoptotic cells, since the in vivo response of gingival tissue 24 h after CHX mouth rinse could be influenced by compensation mechanisms aimed to rescue cells from death. Instead, we were more interested in exploring the potential pathways activated by CHX in vivo. As for apoptosis, we chose to evaluate the involvement of p53/BAX pathway. In fact, previous findings demonstrated that BAX is a p53 downstream effector [42]. Some data reported the centrality of BAX in this pathway, demonstrating that BAX-deficient cells were protected from p53-induced apoptosis [43]. On the other hand, although caspase 3 has been also defined as an enzyme with an important role in the initiation of apoptosis [44], the occurrence of BAX-mediated apoptosis in a caspase-independent manner has been reported [45]. Therefore, BAX expression seems to be more relevant than caspase 3 activation. Moreover, while activated caspase 3 could have been assessed only by IHC, more accurate qRT-PCR methods can be used for the evaluation of BAX expression.

In our results, we did not observe a significant increase in the percentage of stained nuclei after CHX treatment through IHC analysis using p53 as a marker of apoptosis. However, we can infer a proapoptotic potential of CHX since we demonstrated a consistently higher expression of the proapoptotic gene BAX in the three enrolled patients. p53 is known to accumulate in the nucleus following death stimuli, such as oxidative stress and genotoxic injury, and to induce activation of downstream proapoptotic gene expression, e.g., PUMA, Noxa, and/or BAX, to induce cell death. Nevertheless, it has been suggested that other kind of injuries can also produce BAX activation members, thus initiating a p53-independent apoptosis [46].

Thus, our results confirmed in vivo the detrimental effect of CHX in reducing cell viability and led us to hypothesize that CHX mouth rinse could trigger a p53-independent apoptosis.

It is known that the mechanism of apoptosis derives from the local environment of proapoptotic cells and it has been reported that in oral wound healing the intrinsic apoptotic pathway predominates, generally initiated by DNA damage, growth factor levels, or cytokine reduction [40]. Interestingly, a study demonstrated that the timing of the peak of gene expression related to intrinsic apoptosis in oral wound healing was most commonly seen at 24 h, and the authors also suggested a correlation between the apoptosis peak and the resolution of the inflammation, both occurring at the same time [39].Thus, it would be expected to observe P53 positive stained nuclei in both groups in our work, as it may be related to the normal intrinsic apoptotic response. However, as mentioned above, this is not correlated with BAX gene upregulation observed in the CHX group, suggesting a different pathway activation.

The increase of cell proliferation during early wound healing is thought to be regulated by a decrease of apoptosis. Instead of this, cellularity reduction during final wound maturation may be controlled by an increase of apoptosis [47]. CHX treatment induces this latter response, but in a very early stage, in which cell proliferation and viability are required for rapid tissue repair.

Fibroblasts become activated upon wounding, as evidenced by expression of αSMA, proliferation and migration to the wound area, and ECM deposition [48].

In our previous studies [28,29], we demonstrated a downregulation of αSMA and Col1a1 in gingival tissue 24 h after injury, in line with clinical observation of reduced scar formation in this tissue. Instead of this, the alveolar mucosal (M) tissue showed the opposite response, according to the clinical observation of scar tissue repair. We observed that CHX-treated G tissue presents similar behavior to M tissue suggesting that it could induce a “fibrotic response”.

The effect of CHX on collagen production was reported by Mariotti and Rumpf [19]. The authors postulated that, at concentrations which have little effect on cellular proliferation, it can significantly reduce both HGF collagen and non-collagen protein production. Consistent with these findings, a very recent study showed decreased COL1 expression after CHX treatment [24]. Here, we observed the opposite response, and this could be related to the differences between in vitro/in vivo analysis [26]. It is noteworthy that these features are similar to those reported in adult skin fibroblasts, which show a reduction in genes associated with proliferation and an enrichment for GO term ECM production and remodeling—related with increasing age [49]. Additionally, it is interesting to mention that CHX intraperitoneal injection has been reported as the most commonly used method to create a peritoneal fibrosis animal model showing increased expression of transforming growth factor β1 (TGF- β1), αSMA, type I collagen, and vascular endothelial growth factor (VEGF) [50].

Based on our group’s previous results [29], it was still interesting to further investigate the findings based on previously assessed genes related to scar wound healing. Through qRT-PCR analysis, we evaluated the expression of RAC1, TIMP1, and SERPINE1 genes. Of note, we observed that gingival tissue after CHX treatment presents the same pattern observed in alveolar mucosal-derived fibroblasts [29], showing RAC1 downmodulation and TIMP1 and SERPINE1 upregulation. These results are in line with the evidence of an increase in collagen deposition mediated by CHX mouth rinses. Moreover, we previously hypothesized that myofibroblast differentiation in gingival tissues is independent of SERPINE1 and TIMP1 expression, and that other pathways could be involved, since HGFs did not show significant changes in the expression of these genes 24 h after injury [29]. One of the more interesting findings to emerge from this study is that after CHX treatment, these genes present changes in their regulation, with similar characteristics to “fibrotic response” tissues (such as alveolar mucosal tissue). Therefore, CHX appears to induce mechanisms related to impaired wound healing, which are not present in gingival tissues under normal conditions.

It is important to highlight that, although a higher tolerance has been demonstrated of human tissues for antiseptic solutions in vivo compared to in vitro tissue culture [51], in the present study we demonstrated that even after only two mouth rinses with 0.12% CHX, gingival tissue behavior is modified, altering the normal wound healing repair response 24 h after injury.

Undoubtedly, our study presents some limitations since the evaluation was carried out in only three patients and in a single period-time. Moreover, the data obtained here should be run in parallel with a clinical evaluation through an accurate assessment of the healing characteristics [52,53]. Although our results should be extended to solve the aforementioned issues, the in vivo data obtained in the present work confirm previous in vitro findings and provide additional in vivo evidence to understand the potential of CHX to negatively interfere in the early phase of human gingival tissue wound healing. However, because of a small sample size, the results should be cautiously interpreted.

One of the main strengths of this study is that the effect of CHX was evaluated in vivo, through a human biopsy wound model. Although through an in vitro assay a better quantitative analysis can be achieved, without the interference of other in vivo factors [54], surgical wounds present particular conditions to consider, such as vascularization, local and systemic inflammatory responses after injury, mechanical forces affecting tissue repair process, multiple cell layers, and presence of saliva and crevicular fluid. All these features are not present in a monolayer culture, and this could produce relevant changes in the oral tissue response. In fact, we observed some differences between our results and the in vitro performed studies and many similarities with in vivo animal studies performed in other medical fields. Therefore, in vivo evaluations appear to be critical to elucidate the mechanisms impairing the wound healing process after the post-surgical use of CHX mouth rinses.

## 4. Materials and Methods

### 4.1. Ethics Statements

The study protocol (ClinicalTrial.gov-NCT04276129) was approved by Sapienza University of Rome Ethics Committee (Ref.5315-Prot.1066/19). Each participant signed an informed consent in accordance with the Declaration of Helsinki (1975, revised in 2013).

### 4.2. Study Design and Patient Selection

The present pilot study involved three systemically healthy adult patients (mean age 39.3 ± 5.44) who had undergone at least two periodontal surgery procedures and who agreed to be “volunteer” for biopsy collection procedures by signing an informed consent. Patients who underwent antibiotic or anti-inflammatory drug consumption during the previous six months, patients in pregnancy or lactation period, and smokers were excluded from the study.

The subjects were enrolled at the clinical center of the Section of Periodontics, Sapienza University of Rome, Department of Oral and Maxillofacial Sciences. Each patient underwent two surgical procedures and was treated in split mouth design to either post-surgical CHX mouth rinses indication (treatment group-CHX) or non-post-surgical mouth rinses indication (no treatment group-NT).

Biopsies from buccal attached gingiva (G) were harvested 24 h after surgical procedures.

### 4.3. Surgical Procedures and Collection of Human Gingival Tissue Samples

All surgical procedures and biopsies were performed by the same operator (MR). At the end of the surgical procedure, primary closure was obtained at the level of the vertical releasing incisions (VRIs). In the treatment group, 0.12% CHX mouth rinses (15 mL/30 s) were indicated two times/day. Therefore, at the time the biopsy collection, the patients had already performed two mouth rinses with CHX. In the NT group, patients did not perform any mouth rinse after surgery. Twenty-four hours after the surgical procedure, gingival biopsies were harvested at the level of the VRIs with a biopsy punch of 2.0 mm diameter.

The biopsy areas healed by second intention and sutures were removed at 1 week.

### 4.4. Histological Analysis

Gingival biopsies were fixed in 10% neutral buffered formalin and processed for paraffin embedding. Blocks of paraffin were cut at 3 μm thickness using a Leica microtome. Sections were deparaffinized in xylene, rehydrated through graded alcohol series nd stained with hematoxylin–eosin (HE) and trichrome Masson according to standard protocols.

### 4.5. Immunohistochemistry

Immunohistochemistry (IHC) was performed using the automated BOND system (BOND-MAX Fully automated IHC and ISH system, Leica Biosystems Newcastle Ltd., Newcastle Upon Tyne, UK), according to the manufacturer’s instructions. Heat induced epitope retrieval was performed through incubation with BOND Epitope Retrieval Solution (BOND Epitope Retrieval Solution 2 (Cat# AR9640), Leica Biosystems Newcastle Ltd., Newcastle Upon Tyne, UK) for 20 min at 100 °C. Endogenous peroxidase activity was blocked by 3% hydrogen peroxide for 5 min at room temperature. Slides were then incubated with the following primary antibodies for 15 min at room temperature: vimentin (BOND™ Ready-To-Use Primary Antibody Vimentin (V9) (Cat# PA0640), Leica Biosystems Newcastle Ltd., Newcastle Upon Tyne, UK), Col1a1 (Mouse monoclonal antibody (clone 3G3) (cat# sc-293182), Santa Cruz Biotechnology, Inc. Dallas, TX, USA), αSMA (BOND™ Ready-to-Use Primary Antibody Smooth Muscle Actin (alpha sm-1) (Cat#PA0943), Leica Biosystems Newcastle Ltd., Newcastle Upon Tyne, UK), Ki67 (BOND™ Ready-to-Use Primary Antibody Ki67 (MM1) (Cat# PA0118), Leica Biosystems Newcastle Ltd., Newcastle Upon Tyne, UK), p53 (BOND™ Ready-to-Use Primary Antibody p53 (DO-7) (Cat# PA0057), Leica Biosystems Newcastle Ltd., Newcastle Upon Tyne, UK). The detection was performed using BOND Polymer Refine Detection System (Cat# DS9800, Leica Biosystems Newcastle Ltd., Newcastle Upon Tyne, UK) according to the automated IHC protocol. Negative control slides were obtained by omitting the primary antibody.

Sections were analyzed using a Leica microscope coupled to a digital camera. Two independent pathologists, blinded to the treatment, observed the immunostaining and, subsequently, images were captured. The staining intensity for αSMA, vimentin, and Col1a1 was determined using a semi-quantitative score (0, no staining; 1, low staining; 2, moderate staining; 3, strong staining) [32,33]. This evaluation was performed by two independent investigators blinded to the treatment, who observed five microscopic fields for each of the three sections randomly selected for each case using the objective ×20.

Immunohistochemical staining for the nuclear proliferation-associated antigen Ki67 and for p53 was estimated as the percentage of stained nuclei among all nuclei visible in the field. The analysis was performed by two blinded examiners. The number of cells with Ki67/p53-positive nuclei was evaluated in 10 random microscopic fields in each cell preparation and expressed as percentage of Ki67/p53-positive nuclei per optical field.

### 4.6. Quantitative Real-Time PCR (qRT-PCR)

Total RNA from CHX and NT gingival biopsies of the three enrolled patients were extracted using TRIzol reagent (Cat# 15596026, Thermo Fisher Scientific, Carlsbad, CA, USA) following the manufacturer’s instructions, and was reverse transcribed using High Capacity RNA to cDNA Kit (Cat# 4387406, Thermo Fisher Scientific, Carlsbad, CA, USA). cDNAs were then used for amplification of BAX, Col1a1, αSMA, RAC1, SERPINE1 and TIMP1, using the appropriate TaqMan gene expression assay kits (Assay IDs: Hs00180269_m1 (BAX); Hs00164004_m1 (Col1a1); Hs00559403 (αSMA); HS00167155-M1 (SERPINE1); HS01902432_S1 (RAC1); HS01092512_ G1 (TIMP1); Thermo Fisher Scientific, Carlsbad, CA, USA). A total of 2 µl/well of template was added to the sample wells along with TaqMan Universal PCR master mix (Cat# 4305719, Thermo Fisher Scientific, Carlsbad, CA, USA) at a concentration of 1 × and water to a volume of 25 µL/well. Assays were conducted in triplicate on an ABI 7500 Real Time instrument (Thermo Fisher Scientific, Carlsbad, CA, USA) using the following conditions: 50 °C for 2 min, 95 °C for 10 min, and then 95 °C for 15 s, and 60 °C for 1 min, repeated 40 times. Relative quantification was performed using GAPDH mRNA as an endogenous control.

### 4.7. Statistical Analysis

Data were analyzed on Prism 8.0 (GraphPad Software, La Jolla, CA, USA) and are shown as mean ± SD from three independent experiments conducted in triplicate. Two-tailed unpaired Student’s *t* test was used for statistical analysis. *p* value < 0.05 was considered statistically significant.

## 5. Conclusions

The present research was designed to evaluate the in vivo effect of post-surgical CHX mouth rinse in the gingival tissue 24 h after injury. The results of this investigation showed significant changes in the expression of BAX, Col1a1, αSMA, RAC1, SERPINE1, and TIMP1 in CHX-treated gingival biopsies when compared with the NT group. These findings further support that features such as increased collagen deposition, myofibroblast differentiation, and cell apoptosis, as well as reduced cell proliferation, could be relevant for a CHX-induced fibrotic transformation, leading to scar tissue repair. Nevertheless, as the present pilot study was performed in only three patients, further investigation is needed to confirm the data obtained and to define a post-surgical clinical protocol that provides a strategic and personalized use of CHX during the first hours after surgery.

## Figures and Tables

**Table 1:** Immunohistochemical scoring of staining intensity for αSMA, Col1a1, and vimentin.

Patient	IHC Score ^a^					
	αSMA		Col1a1		Vimentin	
	NT	CHX	NT	CHX	NT	CHX
1	0	1	2	3	1	1
2	0	1	1	2	1	1
3	0	1	1	3	1	1

IHC, immunohistochemistry; NT, no treatment group; CHX, chlorhexidine mouth rinse group. ^a^ Staining intensity scores were as follows: 0, no staining; 1, low staining; 2, moderate staining; 3, strong staining [32,33].

## Data Availability

Supporting data of this study are available from the corresponding author upon request.
